# Synthesis of CuO, ZnO nanoparticles, and CuO-ZnO nanocomposite for enhanced photocatalytic degradation of Rhodamine B: a comparative study

**DOI:** 10.1038/s41598-024-60008-7

**Published:** 2024-04-27

**Authors:** M. Jeevarathinam, I. V. Asharani

**Affiliations:** Department of Chemistry, School of Advanced Sciences, Vellore Institute of Technology, Vellore, 632014 Tamil Nadu India

**Keywords:** Environmental chemistry, Environmental chemistry

## Abstract

Water pollution, arising from the presence of toxic dyes and chemicals, is a global challenge, urging the need for eco-friendly solutions in water decontamination. This study focused on the synthesis of copper oxide nanoparticles (CuO NPs), zinc oxide nanoparticles (ZnO NPs), and a bimetallic CuO-ZnO nanocomposite (CZ NC) through an environmentally friendly method employing *Tragia involucrata* L. leaf extract. Comprehensive analysis of structural and optical properties involved using various analytical techniques such as XRD, FT-IR, XPS, UV-DRS, PL, FE-SEM, EDAX, TEM, SAED, zeta potential, TGA, and BET. In comparison to pristine CuO and ZnO NPs, the CZ-NC demonstrated notably enhanced photocatalytic activity in the degradation of Rhodamine B dye (RhB). The optimum conditions for RhB degradation were found to be a pH of 9 and a catalyst dosage of 1 mg/mL for a concentration of 10 ppm. Under these conditions, CuO NPs, ZnO NPs, and CZ-NC demonstrated high efficiencies of 78%, 83%, and 96.1% respectively over 105 min. Through LC-HRMS, the identification of degradation products offered valuable insights into the pathway of photocatalytic degradation. Furthermore, toxicity analysis of intermediates, conducted through ECOSAR software, indicated the formation of non-toxic by-products (ChV/LC_50_/EC_50_ > 100) after the completion of the reaction. Furthermore, the recycled catalysts exhibited sustained stability for up to 4 cycles, with only a minor decrease in activity of up to 6.8%. This confirms their catalytic efficacy in purifying polluted water. This research significantly contributes to the progress of environmentally friendly nanocomposites, enhancing their efficacy in the realm of environmental remediation.

## Introduction

Ensuring the quality of water sources is essential for human life and the overall well-being of Earth's ecosystems. However, changing human lifestyles and industrial expansion have led to water and air pollution, resulting in various health challenges. Recently, increasing environmental concern has focused on hazardous pollutants, specifically dyes originating from the textile industry^1,2^. The widespread use of dyes in textiles has made them significant contributors to aquatic pollution, posing threats to both aquatic life and human health due to their non-biodegradable and carcinogenic properties. Based on recent survey findings, it has been projected that a significant portion, approximately 65%, of the global population will be adversely affected by a scarcity of clean drinking water by the year 2050^2^. Several synthetic organic dyes, such as the xanthene-based Rhodamine B dye commonly used in textiles, display high solubility and possess the potential to induce organ inflammation in living organisms^3^. This toxic textile dye was used as a hue additive in cotton candy and food items and was recently banned by India’s Tamil Nadu government, which could be harmful to people’s health. Despite extensive research efforts, conventional methods such as biological oxidation, adsorption, coagulation, precipitation, and filtration have shown limited efficacy in treating dye pollutants and may produce toxic compounds during the treatment process^4^. Presently, chemical approaches are utilized for treating dye contaminants; however, their application is restricted by significant chemical consumption, pH sensitivity, and the generation of hazardous by-products, including carcinogenic aromatic amines, resulting in increased operational costs^5^. Similarly, electrocoagulation, a prevalent technique for achieving heightened pollution degradation rates, has drawbacks, encompassing rapid depletion of sacrificial anodes, necessitating frequent replacements, potential sludge generation, and diminished treatment efficiency attributed to electrode passivation^6^.

Recent studies indicate the effectiveness of advanced oxidation processes (AOP) such as sonolysis, ionizing radiation, Photo-Fenton, ozonation, and photocatalysis as different methods for removing pollutants from water^7^. Employing metal oxides in photocatalytic methods for breaking down dye pollutants relies on the generation of hydroxyl radicals (^•^OH) and superoxide radicals (^•^O_2_^-^) during the decomposition process. This leads to the production of non-toxic by-products such as water and carbon dioxide, ensuring the success of the remediation process^8–10^. The photocatalytic process is a viable approach for the removal of pollutants from water, presenting a cost-effective solution to address environmental contamination^11^. The photocatalytic effectiveness of metal NPs is influenced by various factors, including particle size and shape^12,13^, surface modifications^14,15^ and surface area^16^. The synthesis of nanoparticles, accomplished through methods such as chemical reduction, co-precipitation, sol–gel processes, and environmentally friendly approaches involving biological agents, provides precise control over size, shape, and surface properties^17^. These versatile nanoparticles find applications in medicine for drug delivery, imaging, and diagnostics, as well as in catalysis, electronics, and environmental remediation^18^. Continued research is exploring their utilization in targeted drug delivery and innovative therapeutic approaches in medicine. This not only contributes to smaller and more efficient components in electronics but also enhances solar cell efficiency and enables environmental remediation^19^. Nanomaterials' multifaceted contributions continue to shape innovations across diverse scientific disciplines^20^.

Distinguishing themselves from various transition metal oxide counterparts, copper oxide (CuO) nanoparticles exhibit commendable catalytic and conductivity properties, characterized by p-type conducting behavior. This behavior stems from their lower bandgap of 1.7 eV, efficient electron transport, non-toxicity, and the presence of a higher number of active sites within their monoclinic structure^21,22^. These attributes find applications across diverse fields, including sensors, optoelectronics, magneto-electronics, and biomedical applications. Bimetallic nanoparticles (NPs) have gained increased attention for their superior properties, driving innovative applications that outperform their monometallic counterparts^23^. In contrast to monometallic alternatives, bimetallic systems offer unique advantages, demonstrating enhancements and modifications across various characteristics. The integration of mechanical, functional, electronic, and structural adjustments results in a synergistic effect when incorporating two disparate metals^24^. These interactions activate controlled optical, thermal, magnetic, plasmonic, and electrical features, significantly broadening their functionalities and applications in catalysis^25^. The improved attributes of bimetallic systems suggest a more effective pathway toward sustainable advancements.

In the realm of photocatalysis, the significance of bimetallic NPs lies in their efficient electron transport between the valence and conduction bands, leading to the generation of more radicals. Incorporating secondary transition metal oxides into CuO NPs has improved the properties of pure copper oxides, affecting the bandgap, conductivity, electrocatalytic, and photocatalytic activity^21,26^.

To further enhance these characteristics, an n-type ZnO semiconductor was chosen for its high chemical stability, outstanding photostability, and widespread availability in society^27,28^. The incorporation of ZnO into CuO nanoparticles creates additional active sites for catalytic activity and suppresses the recombination of electron–hole pairs^29^.

Various methods are employed for synthesizing metal oxide (MO) NPs, categorized as either bottom-up or top-down approaches. Increasing attention is being given to non-toxic, environmentally friendly green synthesis methods that utilize naturally available plants for NPs synthesis^30^. Plant extracts, abundant in phytochemicals, play a crucial role in both the reduction and the stabilization of NPs. Previous studies indicate that the use of plant extracts for metal NPs synthesis is a more dependable approach compared to other biogenic methods^31^. As an example, Aragaw et al. employed *Eichhornia crassipes* plant extract to synthesize a p-Co_3_O_4_/n-ZnO heterojunction photocatalyst, successfully degrading methylene blue dye^32^. Another study by M. Ahmad et al. utilized *Carya illinoinensis* leaf extract to fabricate ZnO and Au-decorated ZnO NPs for the photocatalytic degradation of Rhodamine B^33^.

In this investigation, we have utilized *Tragia involucrata* L known as "Senthatti” for the first time to synthesize CuO NPs, ZnO NPs, and a copper oxide-zinc oxide nanocomposite (CZ-NC). This plant species is renowned for its rich phytochemical composition, encompassing alkaloids, flavonoids, tannins, saponins, steroids, and carbohydrates^34–36^. Therefore, we are fascinated to explore the potential of plant material to synthesize different nanomaterials and study their photocatalytic efficiency against the degradation of RhB, extensively used in the textile and paint industries, leading to significant water contamination due to its toxic and non-biodegradable characteristics. The effect of various parameters on the degradation of RhB was also studied in the presence of CZ-NC. The toxicity of the products resulting from the photocatalytic degradation of RhB was analyzed using the ECOSAR software. The produced products are less toxic compared to the parent dye molecules. A comparison between the present study and the previously reported results on photocatalytic RhB dye degradation is also presented.

## Experimental methods

### Reagents

Copper nitrate trihydrate (Cu(NO_3_)_2_·3H_2_O) and zinc nitrate hexahydrate (Zn(NO_3_)_2_·6H_2_O) were acquired from Sisco Research Laboratory in India. RhB dye (C_28_H_31_N_2_O_3_Cl) was purchased from Central Drug House Pvt Ltd, India. Sodium hydroxide (NaOH) and hydrochloric acid (HCl) were obtained from Molychem, India. Acetonitrile (CH_3_CN) of HPLC grade was supplied by SD Fine Chem Ltd, India. The chemicals were used without additional purification, and double-distilled (DD) water was employed in the NPs preparation process. *Tragia involucrata* L. leaf was locally collected in the Arakkonam region of Tamil Nadu.

### Preparation of *Tragia involucrata* L. leaf extract

The Botanical Survey of India recorded the green source of *Tragia involucrata* L. at Coimbatore (BSI/SRC/5/23/2021/Tech-176). The plant collection and usage adhered to all relevant guidelines. The *Tragia involucrata L*. leaves were cleaned with deionized water to remove dust and then dried in a dark place at room temperature for 7 days before being crushed into a powder. Subsequently, 10 g of the powdered leaves was heated in 100 mL of deionized water for 2 h at 80 °C and filtered. The resulting green solution was then stored in the refrigerator.

### Synthesis of photocatalysts

To synthesize the CuO-ZnO nanocomposite (CZ-NC), 2.41 g of Cu(NO_3_)_2_·3H_2_O and 2.97 g of Zn(NO_3_)_2_·6H_2_O precursors were dissolved in 80 mL of water, and 20 mL of TI leaf extract was added. The mixture was then heated at 80 °C with constant stirring. The pH of the solution was adjusted to 12 using NaOH. After 2 h, the green solution transitioned to a brown color, indicating the successful formation of CZ-NC. The resulting mixture was centrifuged to collect the precipitate, which underwent thorough washing with DD water to remove impurities. Subsequently, the nanocomposite was subjected to calcination at 400 °C to eliminate plant debris. In parallel, pristine CuO NPs and pristine ZnO NPs were prepared separately using the same procedure, utilizing Cu(NO_3_)_2_·3H_2_O precursor for CuO NPs and Zn(NO_3_)_2_·6H_2_O for ZnO NPs. The plant extract plays a vital role in nanoparticle synthesis, serving as both a robust reducing and stabilizing agent. Polyphenolic groups, directly tied to the number of hydroxyl groups, contribute to the extract's reduction potential. Notably, flavonoids within the extract exhibit reduction potentials ranging from 0.119 to 1.021 V^37^.

**Figure 1:**
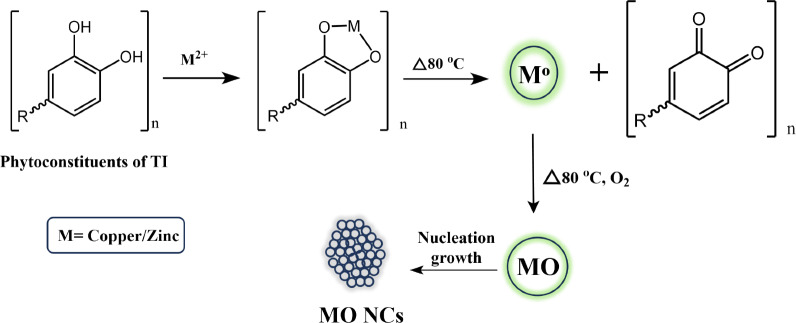
A possible mechanism for the synthesis of nanomaterials.

Figure 1 illustrates a schematic diagram of the interaction between plant extract components and metal ion precursors, elucidating the mechanism of nanoparticle formation. The distinct reduction potentials of CuO (0.34 V) and ZnO (-0.76 V) highlight the redox compatibility governing the efficiency of the reduction process^38^.

### Characterization methods

The synthesized catalyst underwent thorough characterization, including X-ray diffractometry (XRD) using X’–Pert Pro for structural analysis and Fourier-transform infrared spectroscopy (FT-IR) with PerkinElmer to identify functional groups. X-ray photoelectron spectroscopy (XPS) from Thermo Fisher revealed chemical states and elements, while UV–visible (UV–Vis) spectra with JASCO V-670 PC were employed to monitor CZ-NC formation. Scanning Electron Microscopy (SEM) with FEI-Tecnai G2 20 Twin was used to analyze the surface characteristics of CuO and ZnO NPs, and high-resolution transmission electron microscopy was utilized to examine the surface structure, size, and compositions of CZ-NC. The Hitachi F7000 spectrofluorometer was used to estimate the recombination (e^−^/h^+^) performance of the synthesized catalysts. Zeta potential values (Horiba scientific SZ-100) provided insights into stability and surface charge. The pore size and surface area were analyzed using the Brunauer–Emmett–Teller (BET) technique with AutosorbiQ from Quantachrome USA. Ultra Performance Liquid Chromatography (UPLC) from WATERS SM-FTN ACQUITY H-CLASS PDA sensor monitored degradation over time, and Liquid Chromatography-High-Resolution Mass Spectrometry (LC-HRMS) with WATERS–XEVO G2-XS-QToF identified photocatalytic degradation products.

### Photocatalytic dye degradation

The photocatalytic efficiency of the prepared catalysts was assessed by degrading RhB dye (10 ppm) in a UV-light-irradiated photoreactor at room temperature. A mercury vapor lamp (365 nm, 250 W) served as the light source. Initially, a 50 mL RhB dye solution (10 ppm) was stirred with the catalyst (1 mg/mL) for 30 min to attain adsorption and desorption equilibrium. The resulting mixture was then transferred to a quartz tube and exposed to light irradiation under a mercury vapor lamp in an annular-type photoreactor, with continuous stirring facilitated by an air pump. The aliquots (2 mL) were collected at regular time intervals, filtered to remove the catalyst, and analyzed for absorbance using a UV–Vis spectrophotometer. After complete degradation, the catalyst was separated from the solution, dried, and reused following an ethanol-washing treatment. To elucidate the dye degradation mechanism, a similar reaction was conducted with different scavengers such as ethylenediaminetetraacetic acid (EDTA), p-benzoquinone (PBQ), and terephthalic acid (TPA). The percentage (%) of degradation was determined to assess the impact of these scavengers.

## Results and discussion

### Powder XRD analysis

**Figure 2:**
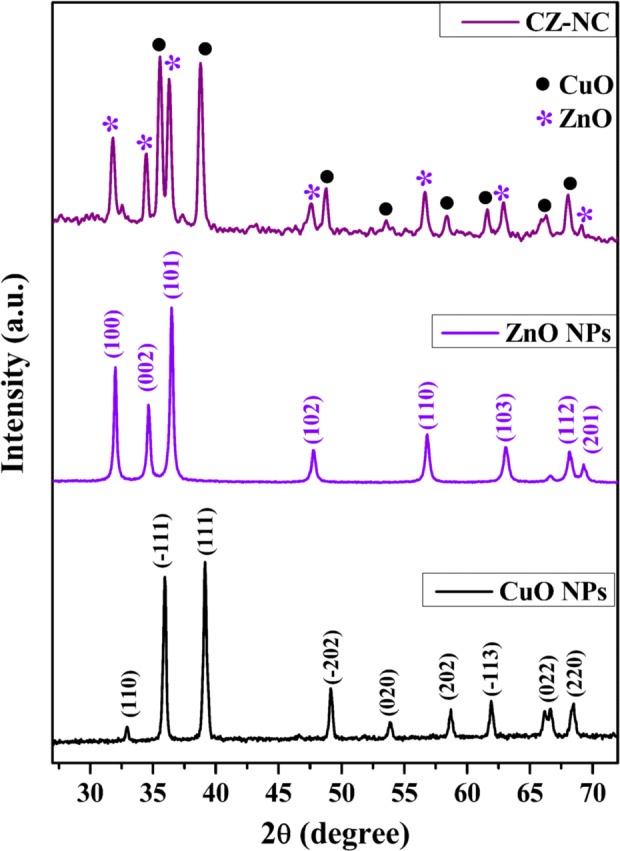
Powder XRD analysis of CuO NPs, ZnO NPs and CZ-NC.

Figure 2 depicts the powder XRD pattern of the prepared CuO NPs, ZnO NPs, and the CZ-NC. XRD analysis aimed to elucidate the structural properties of the prepared nanomaterials. In Fig. 2, the peaks corresponding to CuO and ZnO NPs are noticeable and individually labelled as CuO and ZnO NPs. Concerning CuO NPs, the diffraction peaks at 2θ angles of 35.5°, 38.7°, 48.7°, 53.4°, 58.3°, 61.5°, 66.2°, and 68.1° were identified as reflection planes (110), (111), (-202), (020), and (202)^39^. These crystalline planes strongly confirm the monoclinic structure of CuO NPs, corroborated by comparison with JCPDS No. 05–0661. For ZnO NPs, peaks were observed at 31.7°, 34.4°, 36.2°, 47.5°, 56.6°, 62.8°, and 69.0°, corresponding to (100), (002), (101), (102), (110), (103), and (201) planes^40^. These planes confirm the hexagonal structure of ZnO, consistent with JCPDS No. 036–1451. Furthermore, the XRD pattern indicates the presence of a two-phase CuO-ZnO nanocomposite with no observable impurities. The sharp peaks in the diffractogram suggest the crystallinity of the prepared CZ-NC. The observed pattern aligns with previously reported results, affirming the reliability of the synthesis process^41,42^. The CZ-NC exhibited an average crystallite size of 20.2 nm. In comparison, pristine ZnO and CuO NPs showed average crystallite sizes of 23.3 and 24.1 nm, respectively. Additionally, the presence of two distinct phase structures in the nanocomposite indicates the separate formation of CuO and ZnO NPs. The lattice parameters were calculated and given in Table S1.

### FT-IR analysis

**Figure 3:**
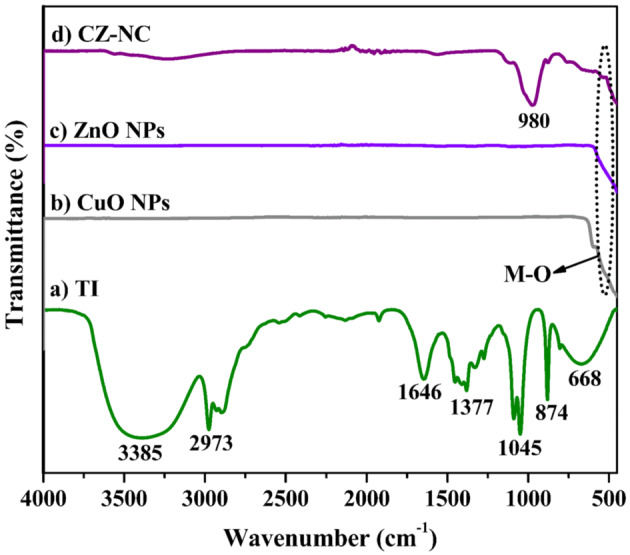
FT-IR spectra of (**a**) TI aqueous leaf extract, (**b**) CuO NPs, (**c**) ZnO NPs and (**d**) CZ-NC.

FT-IR analysis was carried out to identify chemical functional groups associated with the reducing and capping processes during the synthesis of the materials. Figure 3 displays the FT-IR results of *Tragia involucrata L*. (TI) leaf extract, CuO NPs, ZnO NPs, and the CZ-NC, respectively. The functional groups play a crucial role in nanomaterials preparation. Specifically, in TI, the wavenumbers 3385 cm^-1^ and 2973 cm^-1^ were associated with the O–H functional group and C-H alkane, respectively. The bands at 1646 and 1377 cm^-1^ were attributed to C = O stretching and O–H bending vibrations, respectively. Peaks at 1045, 874, and 668 cm^-1^ corresponded to C–O stretching, C–H, and C = C bending vibrations, respectively. These peaks indicate the presence of alkaloids, flavonoids, tannins, saponins, terpenoids, steroids, and carbohydrates in the TI extract^36^. These phytoconstituents' functional groups help in the reduction and stabilization of the nanomaterials. In Fig. 3 b-d, the bands at 513 and 540 cm^-1^ correspond to Cu–O and Zn–O stretching vibrations, respectively. The peaks at 980 and 478 cm^-1^ indicate the formation of CZ-NC^43,44^.

### XPS analysis

**Figure 4:**
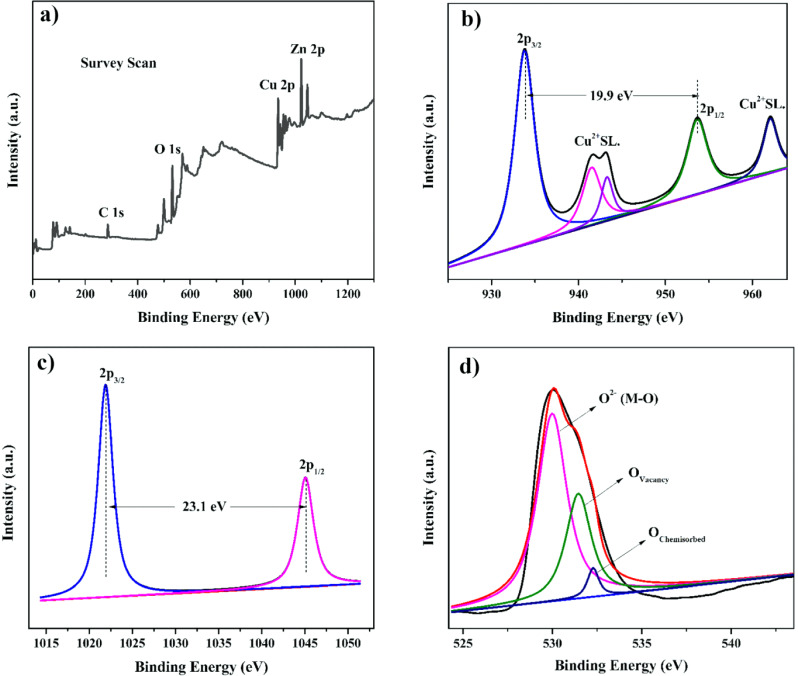
XPS spectra of CZ-NC. (**a**) Overall survey scan, (**b**) Cu 2p, (**c**) Zn 2p and (**d**) O 1 s.

XPS analysis was performed to ascertain the elemental states of the synthesized CZ-NCs. The wide survey spectrum in Fig. 4a reveals the presence of copper (Cu-2p), oxygen (O-1s), zinc (Zn-2p), and carbon (C-1s) atoms in the CZ-NC, with the carbon signal attributed to the plant extract. In Fig. 4b, the XPS of Cu exhibits peaks at 933.8 and 953.6 eV, corresponding to Cu (2p_3/2_) and Cu (2p_1/2_), respectively. The confirmation of Cu atoms in the Cu^2+^ state is evident from satellite peaks at binding energies 941.5 eV and 943.2 eV for Cu (2p_3/2_), along with 962 eV for Cu (2p_1/2_) ^45–48^. Similarly, Fig. 4c displays peaks at binding energies of 1021.9 eV and 1045 eV, indicating the orbitals with doublets 2p_3/2_ and 2p_1/2_, respectively. Additionally, in Fig. 4d, the peak at a binding energy of 530 eV signifies the electron orbital state of O 1s resulting from the oxygen lattice (O^2-^) bonding with metal ions^49^. The peak at a binding energy of 531.5 eV is attributed to O(vac), and the binding energy at 532.3 eV indicates the surface with O-containing groups such as H_2_O, OH, and O^2-^^41,46^. The XPS survey reveals the presence of Cu^2+^O^2-^ and Zn^2+^O^2-^ with specific chemical compositions and states.

### Optical properties

**Figure 5:**
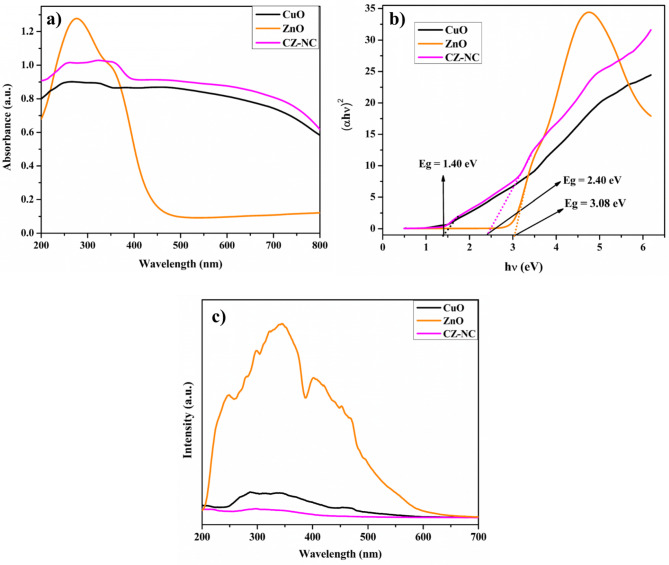
(**a**) UV–Vis DRS of CuO, ZnO NPs and CZ-NC, (**b**) Tauc plot of CuO, ZnO NPs and CZ-NC, (**c**) PL spectra of CuO, ZnO NPs and CZ-NC.

The optical bandgap analysis was conducted to comprehend the optical properties of the synthesized compounds. Figure 5a presents the UV-DRS spectra of ZnO NPs, CuO NPs, and the CZ-NC. The presence of CuO and ZnO NPs is confirmed by the broad absorbance observed in the wavelength range of 200 to 800 nm. The peak observed in the UV-DRS spectrum of the CZ-NC indicates absorption at a wavelength with a red shift towards a longer wavelength region. This shift is associated with the formation of CuO and the incorporation of ZnO, influenced by the smaller band gap of CuO compared to ZnO and the quantum size effect. This shift in absorption wavelength reaffirms the successful formation of the CZ-NC^41,50^. In Fig. 5b, the band gap values for the synthesized CuO NPs, ZnO NPs, and CZ-NC were determined from a Tauc plot.

1 $$\left( {\alpha {\text{h}}\nu } \right)^{\gamma } = {\text{ A}}\left( {{\text{h}}\nu - {\text{E}}_{{\text{g}}} } \right)$$

The absorption coefficient (α), frequency (ν), and γ (indicating whether transitions are direct or indirect, where γ = 2 for direct allowed transitions, γ = 2/3 for direct forbidden transitions, γ = 1/2 for indirect allowed transitions, and γ = 1/3 for indirect forbidden transitions) were considered in determining the energy band gap (E_g_). The calculated energy band gap between the energy bands of the CZ-NC was found to be 2.40 eV. In contrast, CuO NPs and ZnO NPs exhibited energy band gap values of 1.40 eV and 3.08 eV, respectively. This suggests a significantly higher rate of electron transfer in the CZ-NC, potentially leading to increased photocatalytic efficiency.

To examine the process of photogenerated charge carrier (e^-^ and h^+^) separation and recombination, photoluminescence (PL) measurements were taken using an excitation wavelength of 375 nm (Fig. 5c). An intense peak in PL signifies rapid recombination of charge carriers, while a subdued PL intensity indicates a slower recombination probability^41^. CZ-NC displays the least PL intensity compared to other synthesized materials. This indicates that the recombination rate of photogenerated e^-^ and h^+^ is significantly impeded in CZ-NC, suggesting potentially higher photocatalytic activity than CuO NPs and ZnO NPs.

### SEM and EDX

**Figure 6:**
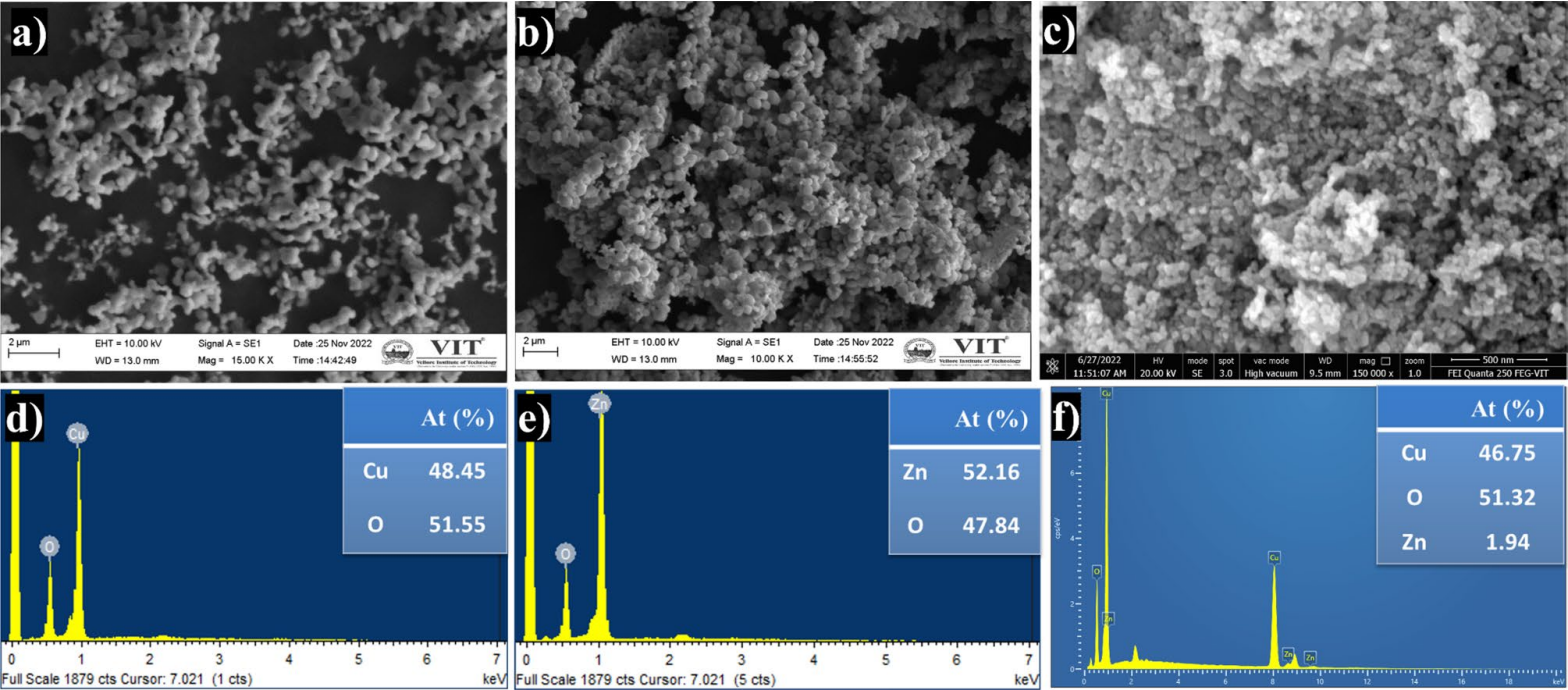
(**a**,**d**) SEM and EDX image of CuO NPs, (**b**,**e**) ZnO NPs and (**c**,**f**) CZ-NC.

The surface characteristics of the synthesized photocatalysts are depicted in Figs. 6a-c. The morphologies of CuO NPs and ZnO NPs are shown in Figs. 6a and 6b, revealing distinctive irregular-sized spherical structures on their surfaces. Figure 6c illustrates the combination of CuO and ZnO, indicating that this combination does not alter the overall structure of the nanocomposite particles. Since zinc ions are incapable of oxidizing copper, the resulting oxides are obtained individually rather than forming a bimetallic oxide. Moreover, the observed particles exhibit homogeneity, suggesting a similar morphology for CuO and ZnO NPs. Notably, CZ-NC demonstrates a higher surface roughness compared to CuO and ZnO NPs. In contrast to pristine CuO and ZnO NPs, the surface of CZ-NC exhibits agglomeration (Fig. 6c), indicating the higher surface energy of CZ-NC^50^.

Figures 6d-f display the EDX spectra, confirming the presence of copper (Cu), zinc (Zn), and oxygen (O) elements with their respective atomic percentages in the synthesized catalyst. Due to its higher reduction potential (0.34) compared to zinc (-0.76), copper undergoes more reduction, resulting in higher atomic ratios in the prepared CZ-NC^38^.

### TEM

**Figure 7:**
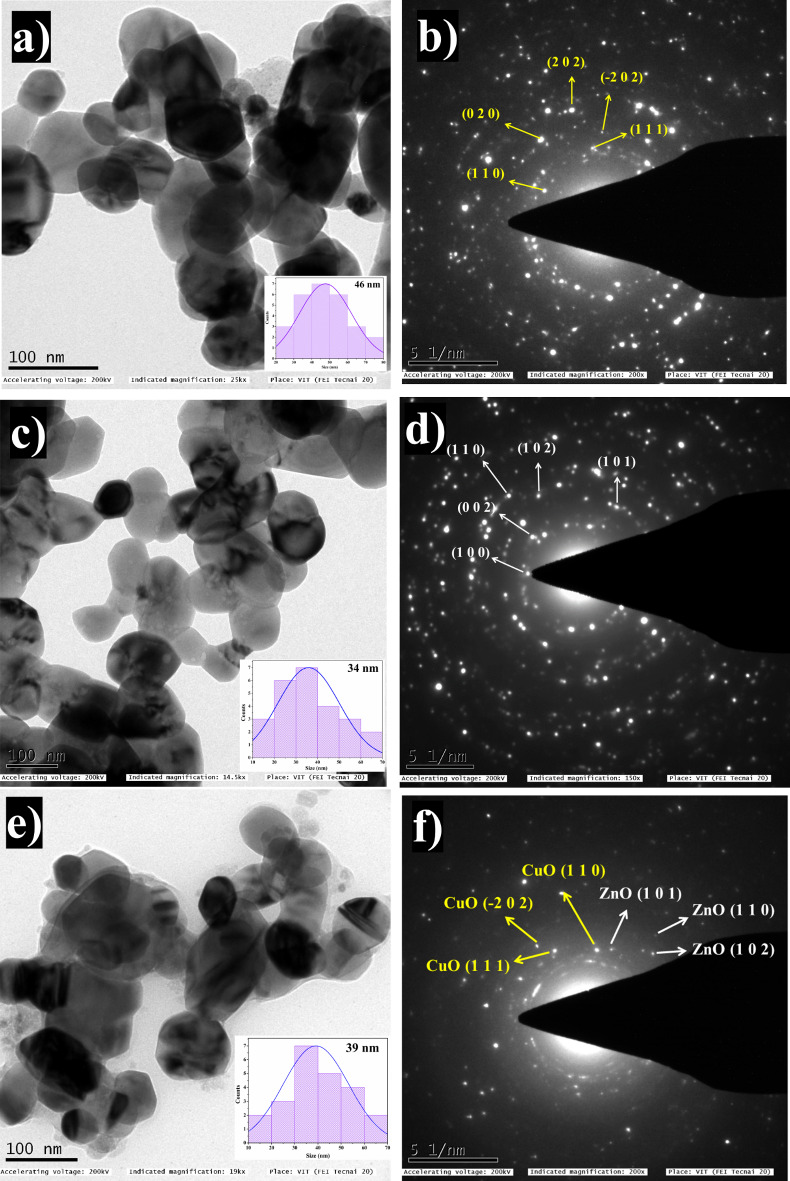
TEM images and SAED patterns of (**a**,**b**) CuO NPs, (**c**,**d**) ZnO NPs and (**e**,**f**) CZ-NC.

The TEM results, depicted in Figs. 7a, 7c, and 7e provide insights into the shape and size characteristics of CuO NPs, ZnO NPs, and CZ-NC. All three—CuO NPs, ZnO NPs, and CZ-NC—display spherical shapes with well-separated particles, maintaining a uniform appearance. This observation is consistent with findings from previous studies where the plant extract-mediated synthesis of nanoparticles resulted in a spherical morphology, demonstrating excellent photocatalytic activity^51^. Through histogram analysis, the calculated particle sizes are determined to be 46 nm for CuO NPs, 34 nm for ZnO NPs, and 39 nm for CZ-NC. The selected area electron diffraction (SAED) patterns in Figs. 7b and 7d reveal lattice plane rings corresponding to CuO NPs and ZnO NPs, displaying successive concentric circles that indicate their highly crystalline nature. In Fig. 7f, the SAED pattern of CZ-NCs shows spots indexed as CuO (111), (-202), and (110), and ZnO (101), (102), and (101). These indexed diffraction rings suggest the presence of CuO with a monoclinic structure and ZnO with a hexagonal structure. The observation of spherical particle shapes, ring patterns, and distinct spots collectively signifies the semi-crystalline nature of the synthesized CZ-NC, consistent with the findings from XRD results^43,52^.

### Zeta potential and BET analysis

**Figure 8:**
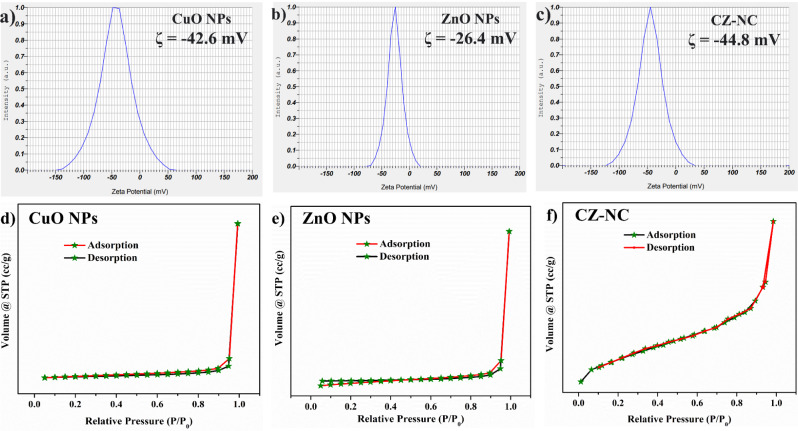
(**a**–**c**) Zeta potential analysis of CuO NPs, ZnO NPs and CZ-NC and (**d**–**f**) BET analysis of CuO NPs, ZnO NPs and CZ-NC.

Figures 8 a-c showcase the results of zeta potential (ZP), providing insights into the surface charges of CuO NPs, ZnO NPs, and CuO-ZnO NCs. The recorded ZP values are -42.6, -26.4, and -44.8 mV for CuO NPs, ZnO NPs, and CZ-NCs, respectively. These values imply electrical repulsion, preventing particle aggregation and ensuring improved stability^53^. The negative ZP indicates the presence of negatively charged ions on the surfaces of NPs, contributing to the enhanced degradation of RhB dye. This is particularly noteworthy as RhB is a cationic dye, promoting better interaction between the dye and the catalyst^54,55^.

The N_2_ adsorption–desorption isotherms for CuO NPs, ZnO NPs, and CZ-NC are illustrated in Figs. 8d-f. This analytical approach is crucial for understanding key factors that influence photocatalytic activity, particularly the direct adsorption of dye onto the catalyst surface. The N_2_ adsorption–desorption isotherm of the nanocomposite exhibits a type IV isotherm characterized by narrow H_3_-type hysteresis loops. The respective surface areas of CuO NPs, ZnO NPs, and CZ-NC were determined to be 7.801, 22.387, and 8.017 m^2^/g. Furthermore, the porosity, as evaluated using the Barrett–Joyner–Halenda (BJH) pore size, was measured at 1.424, 1.420, and 2.028 nm for CuO NPs, ZnO NPs, and CZ-NC, respectively. These results suggest that, compared to CuO and ZnO NPs, CZ-NC exhibits a mesoporous nature with the potential to significantly enhance photocatalytic efficiency^56,57^.

## Photocatalytic experiment

### Photocatalytic RhB degradation

**Figure 9:**
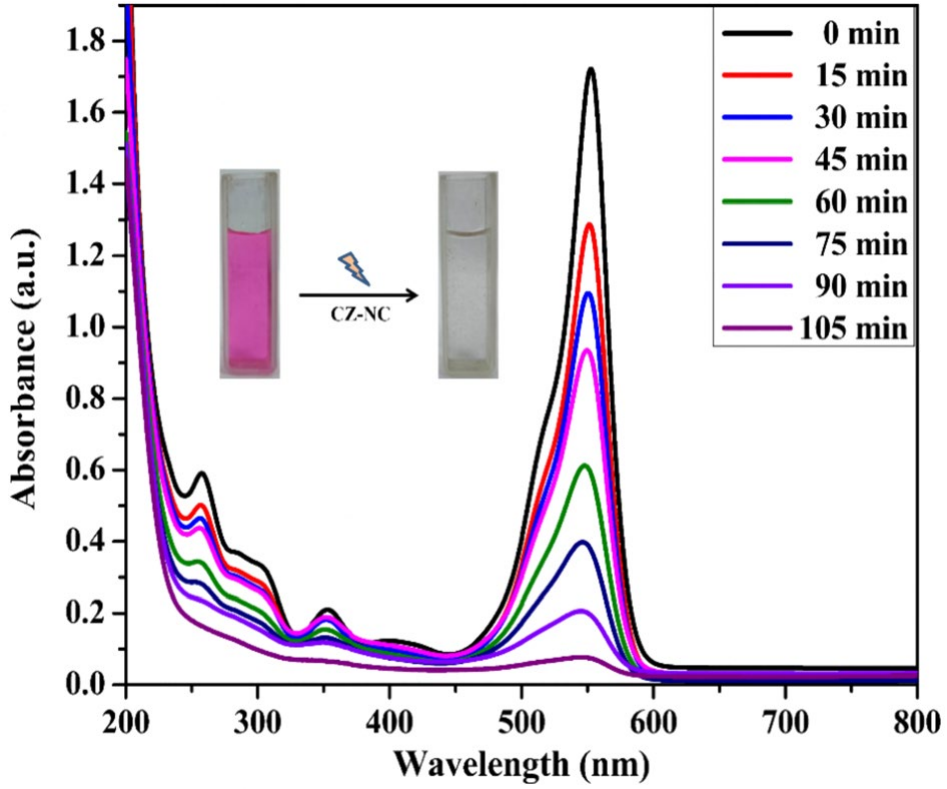
Kinetics of degradation of RhB dye by CZ-NC.

The photocatalytic efficiency of CuO NPs, ZnO NPs, and CZ-NC was assessed by degrading RhB dye under ultraviolet light exposure over 105 min, as depicted in Fig. 9. Regular monitoring of the degradation process using a UV–Vis spectrophotometer revealed a decrease in absorbance intensity at the wavelength of 554 nm. Using Eq. (3), the calculated degradation efficiencies were 78%, 83%, and 96.1% for CuO NPs, ZnO NPs, and CZ-NC, respectively. It may be due to the combined effect of CuO and ZnO in the CZ-NC which significantly enhanced the degradation of RhB. Importantly, in the absence of a catalyst in the reaction, the degradation of RhB was not observed. Hence, the influence of various parameters on RhB dye degradation was investigated exclusively in the presence of CZ-NC. This exploration involved altering the concentration of RhB, the catalyst, and the pH of the reaction mixture.

2 $$\% {\text{ D }} = \, \left( {{\text{C}}_{0} - {\text{C}}_{{\text{t}}} } \right)/\left( {{\text{C}}_{0} } \right)$$

where, C_o_—is the initial absorbance, C_t_—the final absorbance after degradation. The reaction rate was calculated using the relation followed by (4).

3 $${\text{ln }}\left( {{\text{C}}/{\text{C}}_{0} } \right) \, = \, - {\text{kt}}$$

### Influence of pH

**Figure 10:**
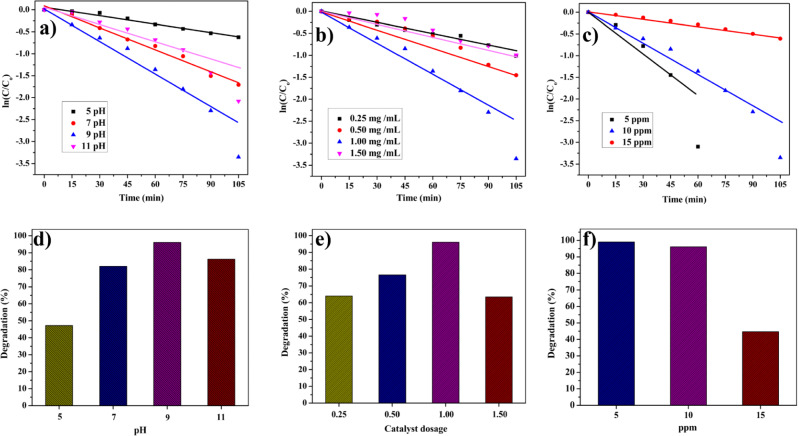
Kinetics plot of degradation of RhB dye at (**a**) different pH, (**b**) different catalyst dosages, (**c**) different concentrations of RhB. The degradation percentage of RhB dye at (**d**) different pH, (**e**) different catalyst dosages, and (**f**) different concentrations of dye.

The catalytic process is notably affected by the pH of the dye solution, a pivotal factor in modifying carriers on the catalyst surface and influencing interactions with hazardous contaminants. To examine the pH effects on the degradation of RhB (10 ppm) in the presence of CZ-NC (1 mg/mL), the pH of the dye solution was maintained as 5, 7, 9, and 11 respectively through the addition of HCl and NaOH. As depicted in Figs. 10 a and d, the relationship between pH and the degradation of RhB dye in the existence of the catalyst under ultraviolet light is evident. The degradation percentages at pH 5, 7, 9, and 11 were recorded as 47.19%, 82%, 96.1%, and 86.2%, respectively, as outlined in Table 1. RhB degradation was limited to pH 5, it may be due to the repulsion between the catalyst and the dye caused by the presence of RhB^+^ ions. Conversely, RhB degradation increased with higher pH values, likely attributed to the deprotonation of RhB^+^ ions at basic pH, resulting in the formation of a Zwitter ion. However, at pH 11, degradation decreased, possibly due to an excess of OH^-^ ions covering the catalyst surface, creating a negatively charged surface that repelled the reaction mixture^58,59^. Therefore, the optimal pH value for RhB dye degradation was determined to be 9.

**Table 1:** Effect of various parameters on the photocatalytic degradation of RhB dye in the presence of CZ-NC.

Parameters	Variations	% of deg	Rate constant (k) (× 10^–4^ s^−1^)
pH	5	47.19	1.0666
	7	82	2.7766
	9	96.1	4.8333
	11	86.2	3.1233
Catalyst dosage (mg/mL)	0.25	64.06	1.4266
	0.50	76.5	2.2983
	1.00	96.1	4.8333
	1.50	63.5	1.6816
Concentrations of dye (ppm)	5	100	8.1733
	10	96.1	4.8333
	15	44.69	0.9650

### Effect of catalyst dosage

Similarly, the impact of catalyst dosage on dye degradation was investigated. Different solutions were prepared with varying catalyst dosages of 0.25, 0.50, 1, and 1.5 mg/mL while maintaining a constant RhB concentration of 10 ppm at pH 9. The observed degradation percentages for the dye were 64.06%, 76.5%, 96.1%, and 63.5%, respectively. Figure 10b,e display the influence of catalyst dosage on the photocatalytic dye degradation process. The results indicate an escalation in dye degradation with an increasing dosage of active catalyst sites. However, a decline in degradation is noted at a 1.5 mg/mL catalyst dosage due to light dispersion and reduced light interaction caused by the higher catalyst quantity, resulting in a more turbid mixture. Additionally, the catalyst surface may aggregate, becoming less accessible to photon absorption, potentially diminishing degradation efficiency. Consequently, the optimal catalyst dosage for RhB dye degradation was found to be 1 mg/mL.

### Effect of concentration of dye

The experiments were conducted separately at varying concentrations of the dye (5, 10, and 15 ppm), with a constant catalyst dosage of 1 mg/mL and a pH of 9. In Fig. 10c,f, the photocatalytic degradation of RhB dye is depicted, showing degradation percentages of 100%, 96.1%, and 44.69% at concentrations of 5, 10, and 15 ppm, respectively. The corresponding rate constants are detailed in Table 1. The observed trend indicates a decrease in the reaction rate as the dye concentration increases. This is attributed to the elevated amount of dye adsorbed on the catalyst surface, limiting both catalyst efficiency and light absorption on its surface.

### Effect of scavengers on the reactive species and mechanism of photocatalytic activity

**Figure 11:**
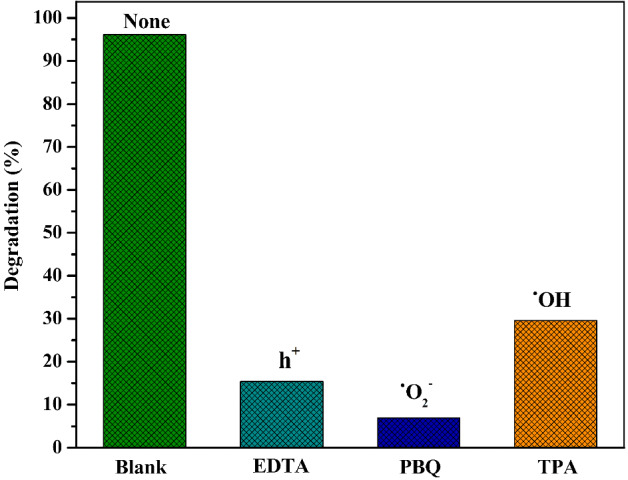
The degradation efficiency of RhB with scavengers.

To identify the reactive species responsible for the degradation process, similar reactions were conducted using various scavengers such as ethylenediamine tetra-acetic acid (EDTA), p-benzoquinone (PBQ), and terephthalic acid (TPA). Each reaction was carried out separately with different scavengers: EDTA for h^+^, PBQ for ^•^O_2_^-^, and TPA for ^•^OH. This approach aimed to assess the specific reactive components involved in the degradation of RhB. Additionally, a blank reaction was performed without any scavengers to understand the impact of scavengers on the reactions. In Fig. 11, the degradation rate was found to be 96.1% without the addition of any scavenger (blank). In the presence of scavengers, the degradation rates were 15.38%, 7%, and 29.57% for EDTA, PBQ, and TPA, respectively. This suggests that ^•^O_2_ is a crucial reactive species for the degradation reaction, although h^+^ and ^•^OH species may also play significant roles in RhB degradation.

According to the results, the following mechanism can be derived for the photocatalytic RhB dye degradation.

4 $${\text{CuO}} - {\text{ZnO }} + hv \to {\text{CuO}} - {\text{ZnO }}\left( {{\text{e}}^{ - } + {\text{ h}}^{ + } } \right)$$

5 $${\text{CuO}} - {\text{ZnO }}({\text{e}}^{ - } + {\text{h}}^{ + } ) \to {\text{CuO }}({\text{h}}^{ + } + {\text{ VB}}) \, + {\text{ ZnO }}\left( {{\text{e}}^{{ - }{}} + {\text{ CB}}} \right)$$

6 $${\text{h}}^{ + } + {\text{ OH}}^{ - } \to^{ \cdot } {\text{OH}}$$

7 $${\text{e}}^{ - } + {\text{ O}}_{{2}} \to^{ \cdot } {\text{O}}_{{2}}^{ - }$$

8 $${\text{H}}_{{2}} {\text{O}} +^{ \cdot } {\text{O}}_{{2}}^{ - } \to {\text{OOH}}^{ \cdot } + {\text{ OH}}^{ - }$$

9 $${\text{2OOH}}^{ \cdot } \to {\text{ O}}_{{2}} + {\text{ H}}_{{2}} {\text{O}}_{{2}}$$

10 $${\text{H}}_{{2}} {\text{O}}_{{2}} +^{ \cdot} {\text{O}}_{{2}}^{ - } \to^{ \cdot } {\text{OH }} + {\text{ OH}}^{ - } + {\text{ O}}_{{2}}$$

11 $$^{ \cdot} {\text{OH }} +^{ \cdot } {\text{O}}_{{2}}^{ - } + {\text{ RhB dye }} \to {\text{ Intermediates }} \to {\text{CO}}_{{2}} + {\text{ H}}_{{2}} {\text{O}}$$

The band edge position of the valence band (VB) conduction band (CB) potential of the synthesized photocatalyst can be theoretically calculated according to the following equation.

12 $${\text{E}}_{{{\text{VB}}}} = {\text{ X }} - {\text{ E}}_{{\text{e}}} + \, 0.{\text{5E}}_{{\text{g}}}$$

13 $${\text{E}}_{{{\text{CB}}}} = {\text{ E}}_{{{\text{VB}}}} - {\text{ E}}_{{\text{g}}}$$

The catalyst's valence band (E_VB_) and conduction band (E_CB_) were identified, with E_e_ representing the energy of free electrons on the hydrogen scale, fixed at 4.5 eV vs. NHE. When evaluating CuO and ZnO NPs, each with electronegativity values of 5.81 eV and 5.79 eV, respectively, the considered parameters included the band gap energy (E_g_) and X, the geometric mean of Mulliken electronegativity^60^.

**Figure 12:**
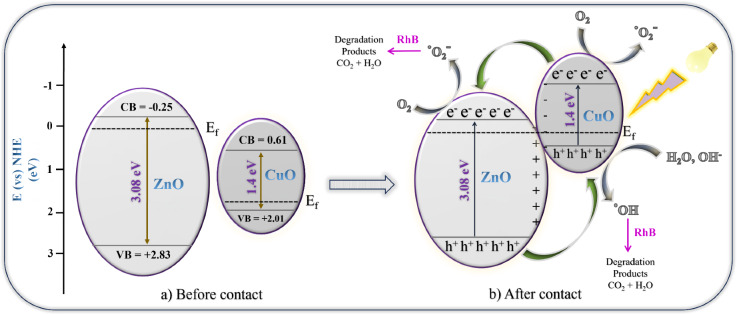
Schematic representation of energy band diagram of CZ-NC (**a**) Before contact and (**b**) after contact of the CZ-NC catalyst.

The calculated energy bandgaps for CuO and ZnO NPs revealed E_VB_ and E_CB_ values of 2.01 eV and 0.61 eV for CuO, and 2.83 eV and -0.25 eV for ZnO, respectively (refer to the Supplementary Material for detailed information). In Fig. 12, the band alignments of CuO and ZnO are depicted both before and after the formation of the nanocomposite. Initially, the conduction band edge and Fermi level of CuO are lower than those of ZnO. As the CuO-ZnO nanocomposite evolves, a discernible shift in Fermi levels occurs. Specifically, the Fermi level of CuO rises, and that of ZnO decreases until equilibrium is achieved, as illustrated in Fig. 12b after contact. This shift is attributed to the transfer of electrons between p-type CuO and n-type ZnO elements. During this electron transfer, ZnO loses electrons, generating a depletion layer on its surface and resulting in a positive shift in the Fermi level. Conversely, CuO gains electrons, leading to a negative shift in its Fermi level. Ultimately, the Fermi levels of both semiconductors equalize. Simultaneously, as these Fermi level adjustments occur, the entire energy band of ZnO lowers, while that of CuO rises^61^. Consequently, in the nanocomposite, CuO possesses a higher conduction band edge than ZnO. Utilizing the equations, experimental results, and information from reported articles, the energy band diagram of the CuO-ZnO nanocomposite is schematically presented in Fig. 12.

## Degraded RhB dye analysis

### UPLC Analysis

The degradation of RhB dye was investigated using UPLC with a 60:40 v/v ratio of water (H_2_O) and acetonitrile (ACN) as the mobile phase. The analysis was conducted at different time intervals (0, 35, 75, 105 min) on RhB dye solutions undergoing photocatalytic degradation. To ensure effective separation, an isocratic elution method was employed, utilizing the H_2_O and ACN combination as the mobile phase. In Figures S1a and b, the UPLC analysis results for the photocatalytic degradation of RhB dye in the presence of CZ-NC are presented. The graph shows a distinct decrease in RhB dye intensity over time, indicating significant degradation^62^.

### LC-HRMS analysis and prediction of toxicity

To investigate the degradation mechanism and intermediates of RhB dye, LC-HRMS analysis was conducted (Figures S2-S4). When the photocatalyst absorbs light, it generates radicals that initiate the degradation of RhB dye. The degradation process involves radical reactions such as N-de-ethylation, de-carboxylation, de-amination, de-alkylation, chromophore cleavage, and ring-opening, ultimately leading to mineralization. In Figure S5, the hydroxyl radicals are shown transforming RhB dye into N-de-ethylated RhB, with subsequent radical attacks causing decarboxylation in the molecular structure, followed by deamination. This sustained action results in the generation of fragments, yielding identified products such as 5-amino-2-(2-hydroxybenzyl) phenol (m/z = 215), 9H-xanthene-3,6-diamine (m/z = 212), 4-aminobenzene-1,2-diol (m/z = 125), glutaric acid (m/z = 132), and 4-aminobut-3-enoic acid (m/z = 101). These findings are in line with earlier reports in the literature^63,64^.

After identifying the intermediates resulting from the photocatalytic degradation of RhB dye, ECOSAR software was employed for toxicity analysis. Acute toxicity, associated with short-term exposure, was assessed using Lethal or Effect Concentration (LC_50_/EC_50_) values, while Chronic toxicity, related to long-term exposure, was evaluated using Chronic values (ChV). Indicator species, including fish, Daphnid, and green algae, were utilized, and the ecotoxicity of both RhB dye and its photocatalytic degradation intermediates was estimated in mg/L, as summarized in Table S2. Certain degraded intermediates (S.No. 9–13) exhibited notably low (LC_50_/EC_50_, ChV) values, indicating toxicity to all indicator species. In contrast, the degraded products, such as hydroxylated and ring-opening structures (S. No. 15, 16, 19, 20), demonstrated higher values, suggesting lower harm compared to the parent molecule RhB. Importantly, with sufficient photocatalytic degradation time, these products could undergo detoxification by decomposing and transforming into CO_2_, H_2_O, and NO_3_^-^ and NH_4_^+^ ions.

### Catalysts recyclability and stability

**Figure 13:**
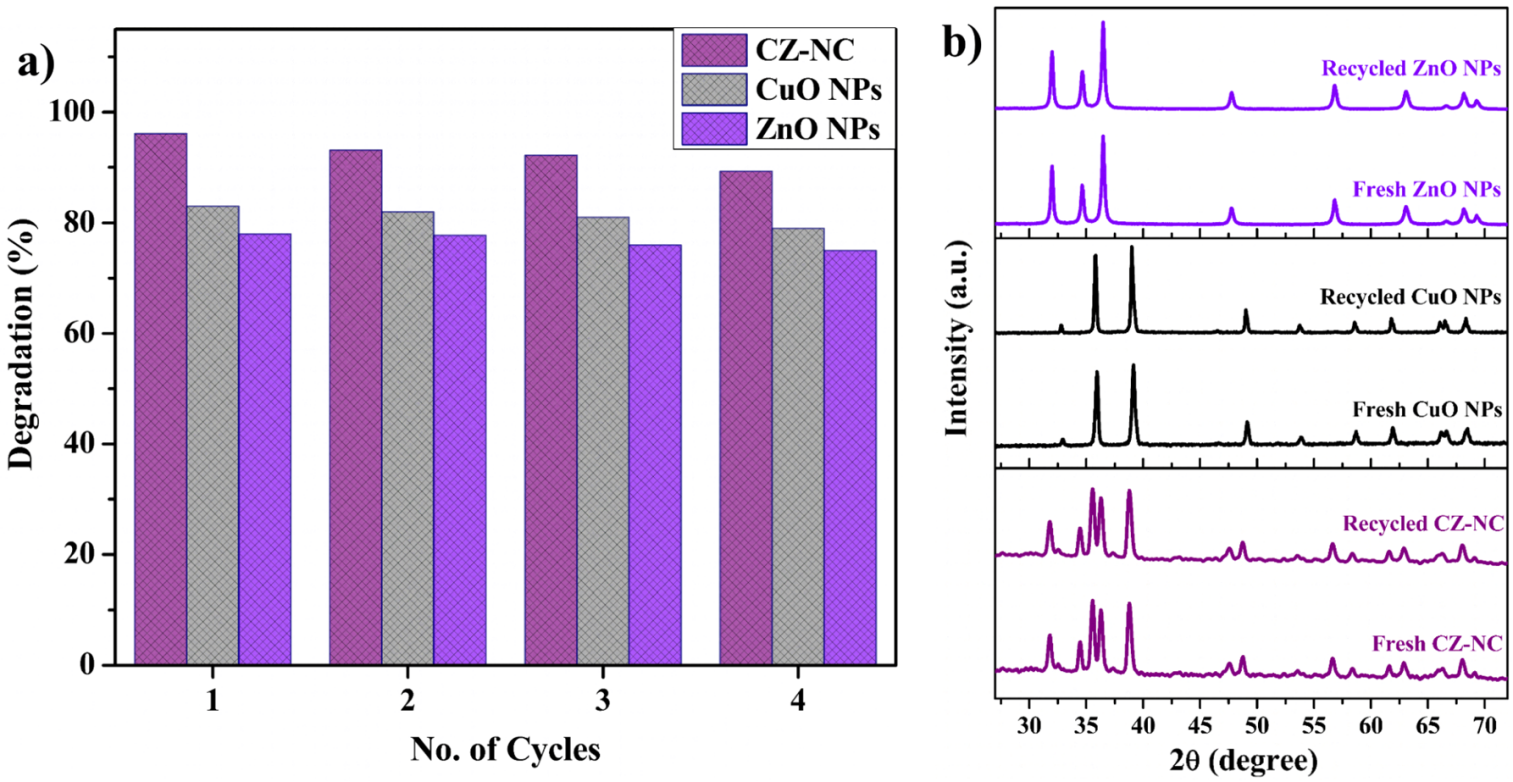
(**a**) Recyclability of CZ-NC, (**b**) XRD spectra of freshly prepared and recycled catalysts after the 4th cycle.

The catalysts were recycled from the reaction mixture separately, undergoing multiple cleaning cycles with water and ethanol before drying to assess stability and cost-effectiveness. Subsequently, the reused catalysts were employed in successive degradation reactions. In Fig. 13a, the degradation efficiency of the catalyst remained consistent for up to four cycles. Figure 13b shows the stability of the recycled catalyst evaluated using XRD. No significant changes were observed in the peak pattern when compared with freshly prepared CuO NPs, ZnO NPs, and CZ-NC. Thus, it demonstrated high stability, making it suitable for the photocatalytic degradation of RhB dye. A comparison between the present study and previously reported results on photocatalytic RhB dye degradation is presented in Table 2. The results indicated that the prepared nanomaterials can be used as a better catalyst for the treatment of textile dye effluent.

**Table 2:** Comparison of the current study with previously reported studies of photodegradation of RhB.

Catalyst	Preparation methods	Dye concentration	Light range	Time	Deg. %	References
Sr/Ce/AC	microwave reduction	20 ppm	Vis	120	76	^64^
CuO	green synthesis	10 ppm	UV	120	91	^65^
ZnO	sol–gel	10 ppm	UV	180	46	^66^
ZnO-Cu_0.5_O	autoclave	10 ppm	Vis	120	73.5	^67^
(CQDs)-doped TiO_2_	sol–gel	5 ppm	UV	120	91.28	^68^
Au-ZnO	green synthesis	10 ppm	UV	180	95	^33^
CuO NPs	green synthesis	10 ppm	UV	105	78	Current work
ZnO NPs	green synthesis	10 ppm	UV	105	83	Current work
CZ-NC	green synthesis	10 ppm	UV	105	96.1	Current work

## Conclusion

This study employed an environmentally friendly method to synthesize CuO NPs, ZnO NPs, and CZ-NC using aqueous leaf extract from TI. The plant-derived phytoconstituents acted as both reducing and capping agents, as confirmed by FT-IR spectroscopy. XRD patterns verified the formation of two-phase CuO and ZnO compounds. Band edge potential calculations for CuO and ZnO suggested significant photocatalytic efficiency in RhB dye degradation for the nanocomposite. CZ-NC displayed remarkable efficiency, achieving 96.1% degradation over 105 min, surpassing CuO NPs (78%) and ZnO NPs (83%) with a calculated reaction rate of 4.8333 × 10^–4^ s^-1^. The identified and analyzed products during photocatalysis indicated the generation of relatively harmless substances. The catalyst exhibited good recyclability, maintaining activity for up to 4 cycles, as confirmed by powder XRD analysis, showing no phase change even after the 4^th^ cycle. Consequently, this catalyst is effective in degrading harmful pollutants, demonstrating its potential application in addressing environmental challenges.

## Supplementary Information

Supplementary Information.

## Data Availability

Data is provided in the supplementary information.
