# Supplementary material for: Synthesis of CuO, ZnO nanoparticles, and CuO-ZnO nanocomposite for enhanced photocatalytic degradation of Rhodamine B: a comparative study
Source: Sci Rep. 2024 Apr 27;14:9718. doi: 10.1038/s41598-024-60008-7 (PMC11577056; doi:10.1038/s41598-024-60008-7)
Supplement: Supplementary file 1 — Supplementary Information. [file 41598_2024_60008_MOESM1_ESM.docx]

**Supporting Information**

**Synthesis of CuO, ZnO Nanoparticles, and CuO-ZnO Nanocomposite for Enhanced Photocatalytic Degradation of Rhodamine B: A Comparative Study**

*Department of Chemistry, School of Advanced Sciences, Vellore Institute of Technology, Vellore 632014, Tamil Nadu, India.

Email: asharani.iv@vit.ac.in

**VB and CB estimation of CuO and ZnO**

Theoretical determination of the valence band (VB) and conduction band (CB) potentials in a semiconductor compound can be achieved through calculations involving Mulliken's electronegativity and the semiconductor's bandgap. These parameters can be assessed using the following equations^1^.

E_VB_ = X − Ee + 0.5E_g_

E_CB_ = E_VB_ − E_g_

In these equations, EVB represents the upper limit of the valence band, E_CB_ denotes the lower boundary of the conduction band in the semiconductor. E_e_ stands for the energy of unbound electrons on the hydrogen scale, maintaining a constant value of 4.5 eV relative to the Normal Hydrogen Electrode (NHE). Additionally, X signifies the geometric mean of Mulliken electronegativity, determined by the constituent atoms in the semiconductor, while E_g_ denotes the energy gap between bands. Mulliken electronegativity for a specific atom is the average of its first ionization energy and first electron affinity.

The step-by-step procedure for computing the valence band (VB) and conduction band (CB) of copper oxide (CuO) is outlined as follows:

The first ionization energy of copper element (Cu): *I_1_* = 745.48 kJ·mol^-1^,

The first electron affinity of copper element (Cu): *E_1_* = 119.15 kJ·mol^-1^,

The Mulliken electronegativity of copper element (Cu)

χ = 1/2(*I_1_* + *E_1_*)

χ = 1/2 (745.48+119.15) = 432.32 kJ·mol^-1^.

Because: 1 eV = 1.6022×10^-19^ C × 1 V = 1.6022×10^-19^ J

And the Avogadro constant (N_A_) = 6.022×10^23^ mol^-1^.

Thus, the Mulliken electronegativity of a copper atom (Cu) can be calculated as follows

χ_Cu_ = 432.32 ×10^3^ J·mol-1 ÷ (6.022×10^23^ mol^-1^) ÷ (1.6022×10^-19^ J)

= 4.480 eV

For oxygen (O): *I_1_* = 1313.94 kJ·mol^-1^, *E_1_* = 140.97 kJ·mol^-1^,

χ = 1/2(*I_1_* + *E_1_*) = 1/2 (1313.94 +140.97) = 727.45 kJ·mol^-1^.

χ_O_ = 727.45×10^3^ J·mol^-1^ ÷ (6.022×10^23^ mol^-1^) ÷ (1.6022×10^-19^ J)

= 7.539 eV

The geometric mean of the Mulliken electronegativity for CuO (*X*) is calculated as follows:
*X* = $\sqrt{{}_{\mathrm{Cu}} {}_{O}}$

*X* = $\sqrt{4.480 7.539}$

*X* = 5.81 eV

The bandgap (E_g_) of CuO was obtained by the Tauc plot, and was determined to be 1.40 eV (Fig. 4 b), i.e., E_g_ (CuO) = 1.40 eV

Therefore, The VB of CuO is calculated as follows:

E_VB_ = *X* − Ee + 0.5E_g_

= 5.81−4.5+0.5(1.40)

= 2.01 eV

The CB of CuO is calculated as follows

E_CB_ = E_VB_ − E_g_

= 2.01-1.40

= 0.61 eV

The methodology used for determining the valence band and conduction band of zinc oxide (ZnO) aligns with a comparable approach. The geometric mean of Mulliken electronegativity for ZnO, denoted as X, is established at 5.79 eV. The resulting values obtained from this process are consistent with those reported in earlier literature^1,2^.

The valence band (EVB) and conduction band (ECB) of zinc oxide (ZnO) were found to be 2.83 and -0.25 eV, respectively.

**Table S1**: The structural parameters of CuO-ZnO nanocomposites

| Phase/Compound | CuO | ZnO |
| --- | --- | --- |
| Lattice parameters (10^-10^ m) | a=4.684  b=3.425  c=5.129 | a=b=3.249  c=5.206 |
| Lattice angles | α= γ=90°  𝛃=99.47° | α= 𝛃=90°  γ=120° |
| Crystal structure | Monoclinic | Hexagonal |
| Cell volume (10^-10^ m)^3^ | 81.16 | 47.62 |
| Calculated density (*ρ*_c_) (g/cm^3^) | 6.51 | 5.675 |
| Space group | C2/c | P63mc |


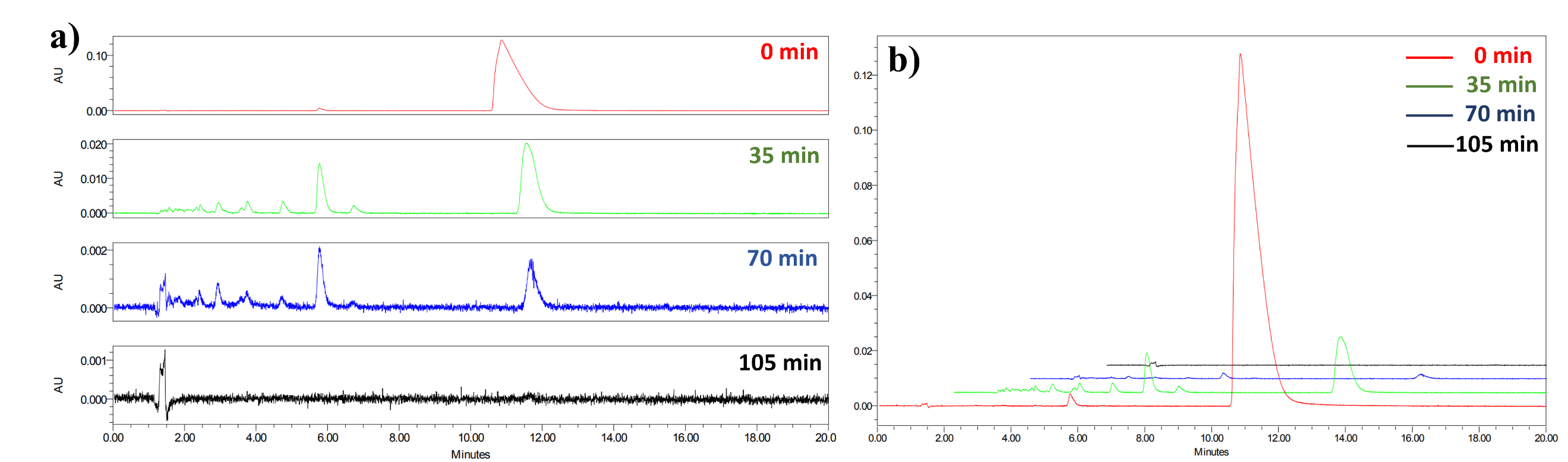


**Fig. S1.** a) UPLC analysis of photocatalytically degraded RhB dye at different time intervals and b) overlay spectra.


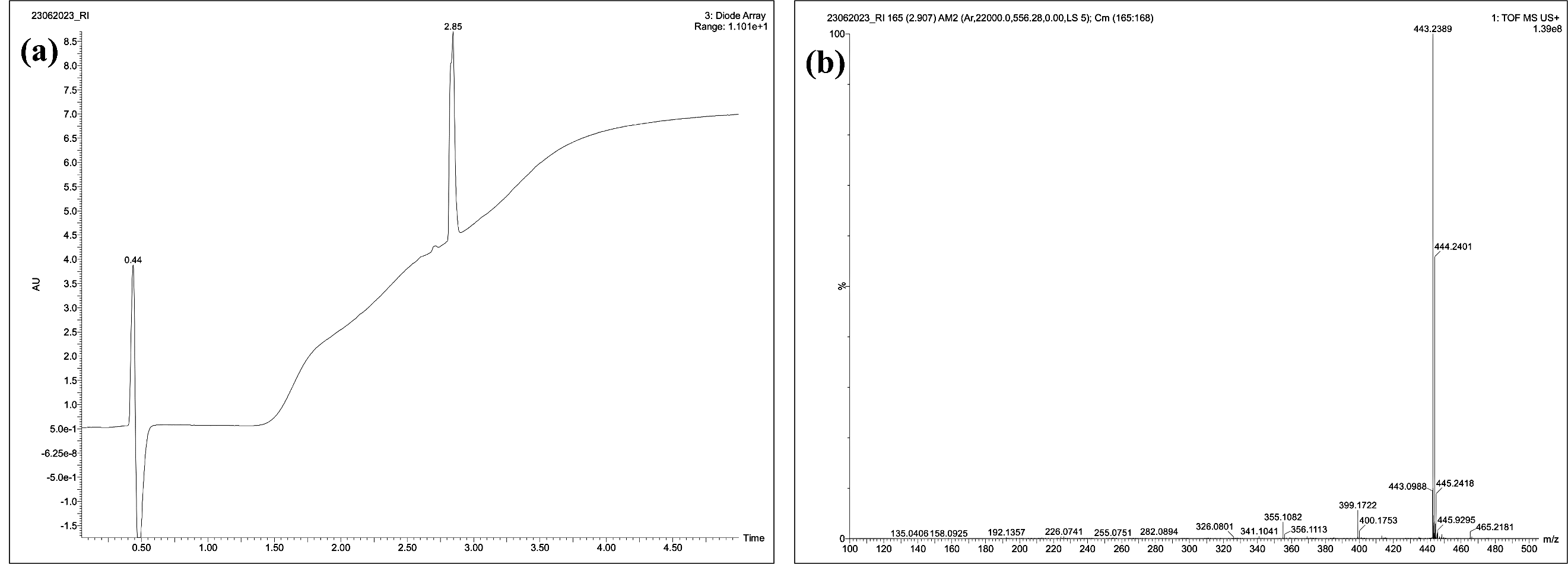


**Fig. S2.** LC-HRMS analysis (a) LC of RhB dye (b) HRMS of RhB dye


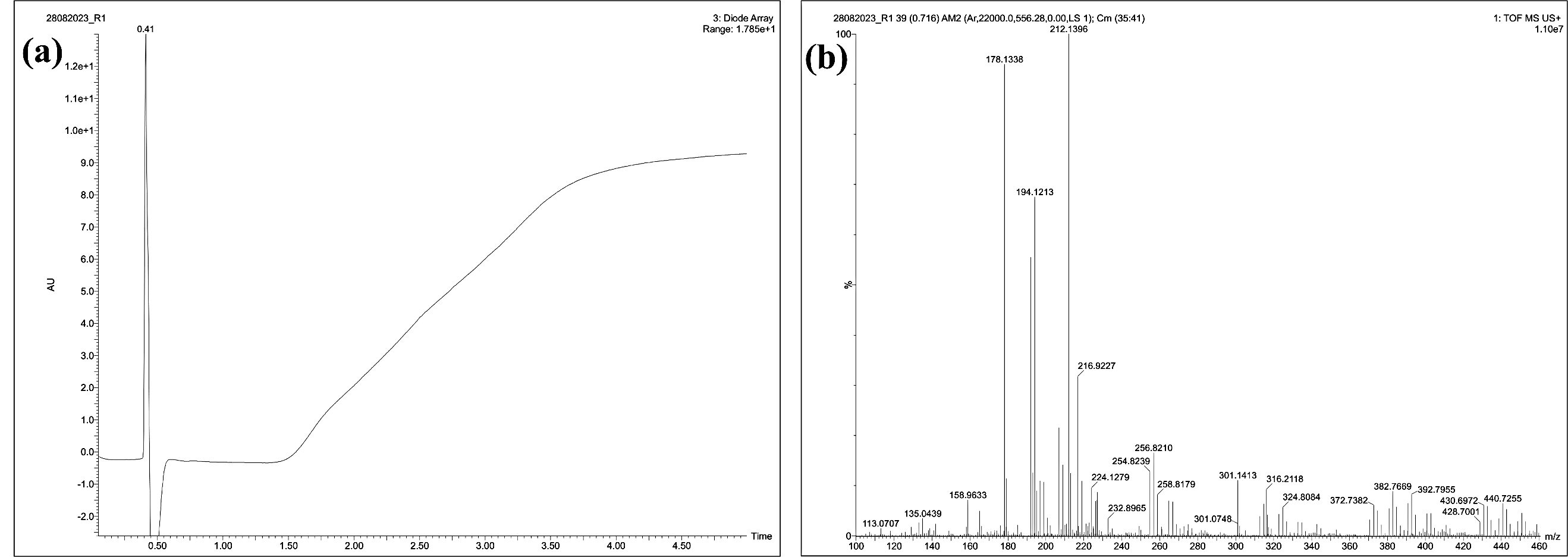


**Fig. S3.** LC-HRMS analysis (a) LC of RhB dye degradation after 50 min (b) HRMS of RhB dye degradation after 50 min.


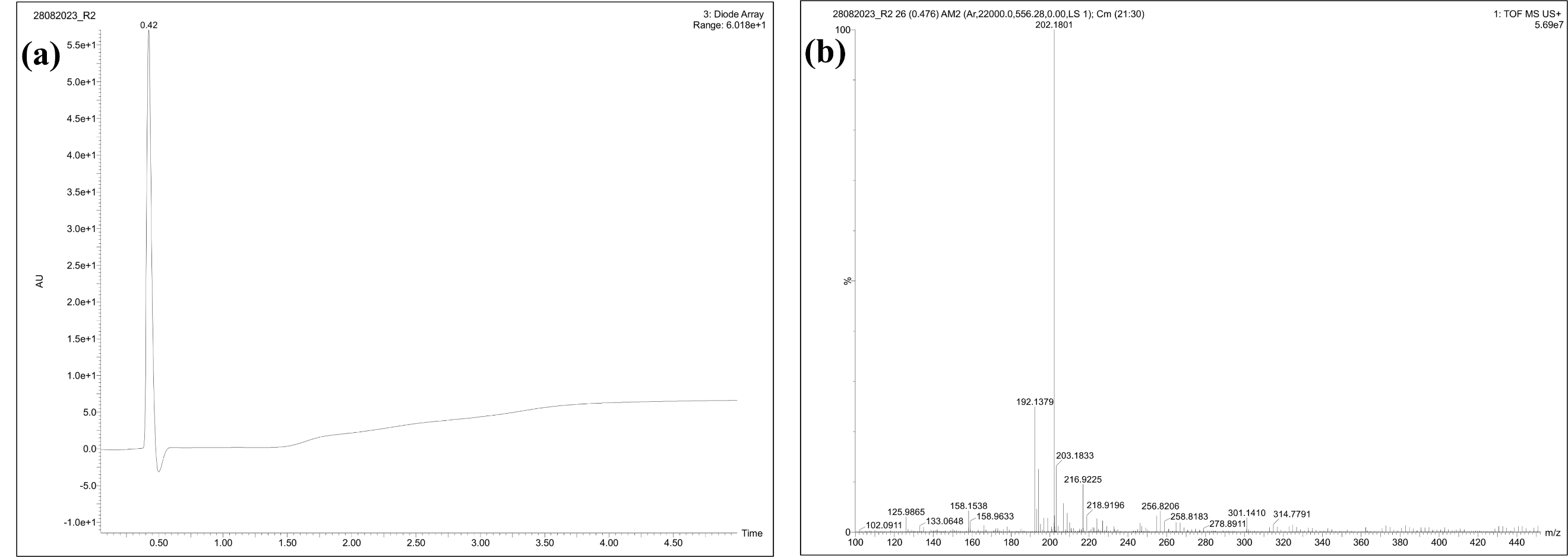


**Fig. S4.** LC-HRMS analysis (a) LC of RhB dye degraded after 105 min, (b) HRMS of RhB dye degraded after 105 min.


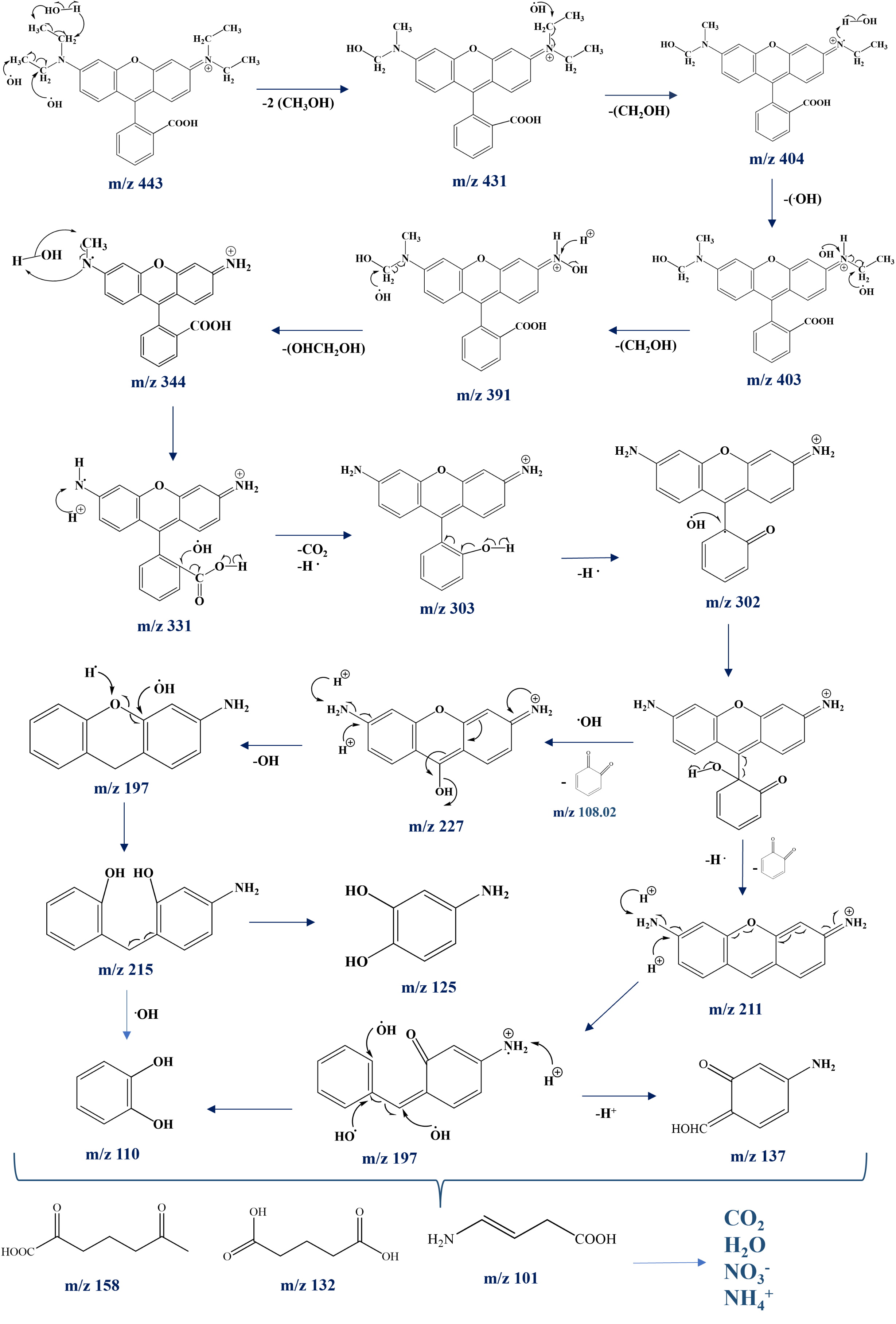

**Fig. S5.** Proposed plausible degradation pathway mechanism of RhB dye.

**Table S2:** ECOSAR-Toxicity analysis of intermediates formed during photocatalytic degradation of RhB.

| **S.No** | **Molecular structure** | **ECOSAR classification** | **Fish (mg/L)** | | **Daphind (mg/L)** | | **Green algae (mg/L)** | |
| --- | --- | --- | --- | --- | --- | --- | --- | --- |
|  |  |  | **LC_50_** | **ChV** | **LC_50_** | **ChV** | **EC_50_** | **ChV** |
| 1 |   Molecular formula: C_28_H_31_N_2_O_3_^+^  m/z: 443 | Vinyl/Allyl/Propargyl Ethers | 1090 | 73.2 | 3550 | 354 | 2720 | 1640 |
| 2 |   Molecular formula: C_26_H_27_N_2_O_4_^+^  m/z: 431 | Vinyl/Allyl/Propargyl Ethers | 25400 | 3270 | 205000 | 20600 | 201000 | 64200 |
| 3 |   Molecular formula: C_24_H_23_N_2_O_4_^+^  m/z: 403 | Vinyl/Allyl/Propargyl Ethers | 5250 | 497 | 27600 | 2760 | 24100 | 10400 |
| 4 |   Molecular formula: C_22_H_19_N_2_O_5_^+^  m/z: 391 | Vinyl/Allyl/Propargyl Ethers | 2410 | 169 | 8770 | 874 | 7160 | 3680 |
| 5 |   Molecular formula: C_20_H_15_N_2_O_3_^+^  m/z: 331 | Anilines (unhindered) | 14400 | 397 | 147 | 1.42 | 586 | 442 |
| 6 |   Molecular formula: C_19_H_15_N_2_O_3_^+^  m/z: 319.11 | Anilines (unhindered) | 2760 | 92.3 | 18.6 | 0.172 | 80.2 | 73.2 |
| 7 |   Molecular formula: C_13_H_11_N_2_O^+^  m/z: 211.09 | Anilines (unhindered) | 1950 | 66 | 12.6 | 0.116 | 54.8 | 50.8 |
| 8 |   Molecular formula: C_13_H_11_N_2_O_2_^+^  m/z: 227.08 | Anilines (unhindered) | 7920 | 387 | 22.8 | 0.194 | 116 | 155 |
| 9 |   Molecular formula: C_13_H_12_N_2_O  m/z: 212.09 | Anilines (unhindered) | 11.2 | 0.093 | 1.66 | 0.021 | 4.00 | 0.896 |
| 10 |   Molecular formula: C_13_H_11_NO  m/z: 197.08 | Anilines (unhindered) | 2.63 | 0.015 | 0.899 | 0.013 | 1.85 | 0.283 |
| 11 |   Molecular formula: C_13_H_10_O_2_  m/z: 198.07 | Phenols | 1.50 | 0.178 | 1.48 | 0.223 | 2.03 | 0.463 |
| 12 |   Molecular formula: C_13_H_13_NO_2_  m/z: 215.09 | Neutral organics | 275 | 26.2 | 153 | 14.1 | 105 | 26.3 |
| 13 |   Molecular formula: C_13_H_12_O_3_  m/z: 216.08 | Neutral organics | 54.1 | 5.63 | 32.4 | 3.66 | 30.1 | 8.86 |
| 14 |   Molecular formula: C_7_H_7_NO_2_  m/z: 137.05 | Vinyl/Allyl/Propargyl Ketones | 2430 | 3540 | 1700 | 231 | 10900 | 130 |
| 15 |   Molecular formula: C_5_H_8_O_4_  m/z: 132.04 | Neutral organics | 117000 | 8940 | 53900 | 2940 | 16900 | 2780 |
| 16 |   Molecular formula: C_6_H_7_NO_2_  m/z: 125.05 | Neutral organics | 10500 | 802 | 4830 | 265 | 1530 | 254 |
| 17 |   Molecular formula: C_6_H_6_O_3_  m/z: 126.03 | Neutral organics | 2070 | 173 | 1030 | 69 | 440 | 85.6 |
| 18 |   Molecular formula: C_7_H_8_O_2_  m/z: 125.05 | Neutral organics | 1860 | 156 | 929 | 63.1 | 405 | 79.4 |
| 19 |   Molecular formula: C_7_H_10_O_4_  m/z: 158.06 | Neutral organics | 127000 | 8620 | 52900 | 2190 | 11100 | 1460 |
| 20 |   Molecular formula: C_4_H_7_NO_2_  m/z: 101.05 | Aliphatic Amines | 1290000 | 589000 | 78100 | 3320 | 246000 | 50100 |

Not harmful: ChV/LC_50_/EC_50_ > 100;

Harmful: 100 ≥ ChV/LC_50_/EC_50_ > 10;

Toxic: 10 ≥ ChV/LC_50_/EC_50_ > 1;

Very toxic: ChV/LC_50_/EC_50_/ ≤ 1 ^3^.

**References**

1. Cheng, Y., He, L., Xia, G., Ren, C. & Wang, Z. Nanostructured g-C_3_N_4_ /AgI composites assembled by AgI nanoparticles-decorated g-C_3_N_4_ nanosheets for effective and mild photooxidation reaction. *New Journal of Chemistry* **43**, 14841–14852 (2019), https://doi.org/10.1039/C9NJ02725D.
2. Xu, Y. & Schoonen, M. A. A. The absolute energy positions of conduction and valence bands of selected semiconducting minerals. *American Mineralogist* **85**, 543–556 (2000), https://doi.org/10.2138/am-2000-0416.
3. Ma, K. *et al.* Degradation and mechanism analysis of chloroxylenol in aqueous solution by gas–liquid discharge plasma combined with ozonation. *RSC Adv* **11**, 12907–12914 (2021), 10.1039/D1RA01886H.
